# Protocol for photoactivation of YAP in cancer cell spheroids embedded in collagen gels

**DOI:** 10.1016/j.xpro.2021.100657

**Published:** 2021-07-07

**Authors:** Bernhard Illes, Hanna Engelke

**Affiliations:** 1 Department of Chemistry and Center for NanoScience (CeNS), Ludwig-Maximilians-Universität München, Butenandtstr. 11, 81377 Munich, Germany; 2 Institute of Pharmaceutical Sciences, Department of Pharmaceutical Chemistry, University of Graz, Humboldtstr. 46, 8010 Graz, Austria

**Keywords:** Cell Biology, Cell culture, Cancer, Microscopy, Antibody, Molecular/Chemical Probes, Biotechnology and bioengineering

## Abstract

This protocol describes the necessary preparations and procedures to photo-activate Yes-associated protein (YAP) with optoYAP in cancer cell spheroids in 3D collagen matrices. We detail steps for immunofluorescent staining of the resulting YAP-activated HeLa spheroids. In addition, we describe handling of optoYAP on 2D substrates. While this protocol focuses on the use of optoYAP in 3D HeLa cell culture, it can be modified for other cell types.

For complete details on the use and execution of this protocol, please refer to Illes et al. (2021).

## Highlights

• Optical control of YAP (Yes-associated protein) activation with optoYAP
• Protocol for embedding spheroids in collagen gels
• Techniques to keep YAP inactivated
• Immunofluorescence staining of spheroids in collagen gels

## Graphical abstract

**Figure:**
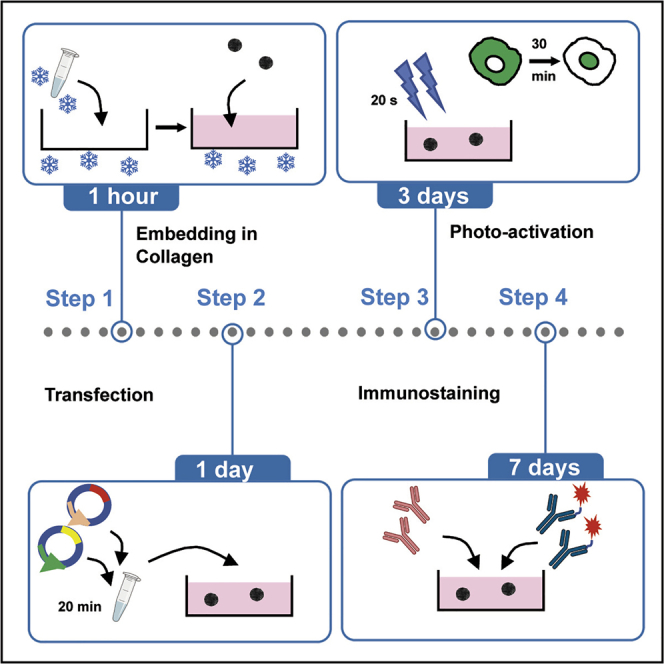

## Before you begin

The protocol below describes the specific steps for photoactivation of Yes-associated protein with optoYAP. Particularly, it describes creating and embedding HeLa cell spheroids in collagen gels, their subsequent transfection with optoYAP plasmids, and the induction of directed growth by photoactivation of optoYAP. Furthermore, it presents an immunostaining procedure for spheroids in collagen gels. This protocol was also used for A431 cells and should also be usable for other cells that allow for the formation of stable spheroids and their transfection.

### Culture HeLa cells

**Timing: 1 week**

1. Thaw HeLa cells at least a week before the planned date of the experiment and culture in DMEM (10% FBS, 1% Penstrep) in an incubator (37°C, 5% CO_2_) until the experiment. Passaging cells twice or more is recommended before starting the spheroid cultures. Passaging more than 20 times before the experiment is not recommended.

### Preparation of HeLa spheroids

**Timing: 1–2 days**

2. Transfer 500 cells in each well of a low adhesion 96-well plate in 100 μL DMEM (10% FBS/1% Penstrep).
3. Let the cells incubate in the low adhesion plate in an incubator (37°C, 5% CO_2_) for 24 h before use. No additional shaking is needed.

***Note:*** This protocol has been optimized for the parameters provided in the respective steps. We therefore recommend sticking to these parameters at least initially. However, if needed, incubation time in low adhesion plates may be varied depending on desired spheroid size and properties. 24 h incubation should result in spheroid diameters of about 250 μm. Longer incubation times will lead to larger and more stable spheroids. Similarly, cell number may be varied depending on desired spheroid size. Less cells will result in smaller spheroids and more cells in larger spheroids. However, changing incubation time or cell number may affect spheroid stability during handling, transfection efficiency, as well as their tendency to sink to the bottom of the gel. Thus, transfection procedure and collagen embedding need to be optimized upon changes in cell number or incubation time of spheroids.

## Key resources table

**Table:** 

REAGENT or RESOURCE	SOURCE	IDENTIFIER
**Antibodies**		

YAP1 polyclonal rabbit antibody	Thermo Fisher Scientific	PA1-46189, RRID: AB_2219137
Donkey anti-Rabbit IgG (H+L) Highly Cross-Adsorbed Secondary Antibody, Alexa Fluor 546	Thermo Fisher Scientific	A-10040, RRID: AB_2534016

**Chemicals, peptides, and recombinant proteins**		

X-tremeGENE™ 9 DNA Transfection Reagent	Sigma-Aldrich	6365779001
Collagen Type I, rat tail	Corning	354249
Formaldehyde	Sigma-Aldrich	252549
Triton X-100	Sigma-Aldrich	X100
DMEM	Thermo Fisher	41965039
PBS	Thermo Fisher	14190185
Photocaged lysine (see note in materials and equipment section)	Synthesized (Gautier et al., 2010; Illes et al. 2021)	n/a
Penicillin/Streptomycin (PenStrep)	Thermo Fisher	15070063
Fetal Bovine Serum (FBS)	Thermo Fisher	10270106
Opti-MEM I	Thermo Fisher	31985062
Bovine Serum Albumin Fraction V (BSA)	Thermo Fisher	15260037
NaOH	Sigma-Aldrich	S2770
Matrigel (optional)	Corning	354234
Bisbenzimid H 33342 (Hoechst)	Sigma-Aldrich	B2261

**Experimental models: cell lines**		

HeLa cells	ATCC	ATCC® CCL-2™

**Recombinant DNA**		

PAG plasmid	Lead contact (Engelke et al., 2014)	Lead contact
OptoYAP plasmid	Lead contact (Illes et al., 2021)	Lead contact

**Other**		

μ-Slide 8 Well	ibidi	80826
Ultra-low adhesion 96-well plate	Corning	7007
UV lamp (Spectroline E-series)	Sigma-Aldrich	Z169625
Inverted microscope Ti-E with pE4000 LED light source	Nikon	n/a

## Materials and equipment

***Note:*** The synthesis of photocaged lysine is described in detail in Gautier et al., 2010; Engelke et al., 2014; and Illes et al., 2021. For storage, the photocaged lysine should be dissolved in water at a concentration of 100 mg/mL and sterile filtered. It can then be stored short term at 4°C (several weeks) and long term (several months) at −20°C. The compound can also be made available from the lead contact upon reasonable request.

***Note:*** For photoactivation, any handheld UV-lamp with sufficient power and 365 nm wavelength (e.g., Spectroline E-Series) or a microscope with DAPI filter or 365 nm laser, which can be regulated to an illumination power of 0.6 mW/mm^2^ can be used. For fluorescence imaging of immuno-stained samples we recommend a microscope with optical sectioning and an objective with long working distance and high N.A.

## Step-by-step method details

### Collagen gel preparation and spheroid transfer

**Timing: 30–60 min**

Preparation of the collagen embedded HeLa spheroids as basis for further experiments in an ibidi 8-well μ-slide.

1. Preparation of the collagen gel mixtures with a total volume of 200 μL per well. Keep everything on ice.
  a. Place 150 μL of a collagen Type I solution (8.35 mg/mL) per well in Eppendorf tube on ice.
  b. Prepare NaOH solution with a mixture of 2.5 μL NaOH (1 M) and 47.5 μL PBS per well in an Eppendorf tube on ice.
  c. Combine both solutions and mix by gently pipetting up and down approximately 3 times before applying 200 μL to each well of an 8-well slide.
2. Carefully collect spheroids and surrounding medium directly from the low adhesion 96-well plate using disposable plastic pipettes and add two of them to each well of your 8-well slide. This should result in a total volume (including collagen solution) of 400 μL in each well. If collection of spheroids did not yield 200 μL of transferred medium, addition of fresh medium to reach 400 μL is recommended.

***Optional:*** Carefully mix the gel after adding the spheroids. (troubleshooting – problem 3)

**Figure 1:**
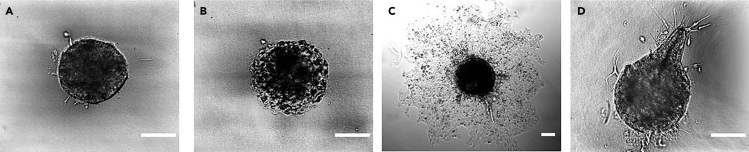
Brightfield images of spheroids in collagen gel (A) Spheroid embedded in collagen before activation of optoYAP. (B) Damaged spheroid in collagen. (C) Invasion of spheroid after photoactivation of optoYAP in the entire spheroid. (D) Site-directed invasion of spheroid after photoactivation of optoYAP on the upper right side. Scale bars: 100 μm. Images were acquired on a Nikon Eclipse Ti-E microscope with a 25**×**, 1.1. N.A. water immersion objective (A, B, D) and on an ImageXpress Micro XLS (Molecular Devices) with a 10**×** objective (C).

3. Place 8-well slide in incubator at 37°C/5% CO_2_ for 30 min for successful gelation.
4. Check presence and shape of embedded spheroids in light microscope. If they are not damaged (see Figure 1 a and b for comparison) proceed to next step. (troubleshooting – problems 1 and 2)

**CRITICAL:** Control of pH of the ensuing gel by addition of the correct amount of NaOH is extremely important as it affects gel formation and may have adverse effects on the cells. Final pH should be 7.4. Also, ensure the collagen stays at low temperatures, as gel formation will immediately start at higher temperatures and affect gel properties and handling**.**

***Note:*** Collagen concentration may vary in different lots, it should be adjusted by varying the volume of added collagen solution to yield a final collagen concentration of 3.13 mg/mL in each well. However, in this case, the amount of NaOH may be necessary to adjust as well to maintain a neutral pH in the final solution. Different final collagen concentrations may be used. However, concentrations below 3 mg/mL may lead to spontaneous invasion and to sinking of the spheroids to the bottom of the slide, which leads to disintegration of the spheroid.

**CRITICAL:** Spheroids should be handled with care avoiding strong shear flow. Thus, we recommend slow pipetting, avoiding narrow pipette tips, and instead using disposable plastic pipettes as described in the protocol steps above. Shear flow may tear spheroids apart or damage them reducing cell viability. Also, pH lower than physiological pH can reduce cell viability. Damage to spheroids (both in terms of shape as well as cell viability) will be visible in the morphology (cf. Figure 1A of an intact spheroid and Figure 1B of a damaged spheroid). However, to ensure cell viability, additional standard viability tests are recommended. The specific test should be chosen such that it does not interfere with the final readout.

### Spheroid transfection

**Timing: 1 day**

Transfection of HeLa spheroids embedded in collagen with optoYAP plasmids.

5. Preparation of the transfection mixture in a 1.5 mL Eppendorf tube. Amounts given per 100 μL total volume, i.e., for 8 wells of an 8-well slide including some excess for losses.
  a. 1 μg optoYAP plasmid
  b. 1 μg PAG plasmid
  c. 3 μL Xtreme Gene 9 transfection reagent
  d. Fill up to a total volume of 100 μL with Optimem
6. Carefully mix by tapping the tube. Incubate for 20 min at room temperature (20°C–25°C).
7. Use 10 μL of transfection mixture per well for an 8-well slide.
8. Add 3 μL of photocaged lysine per well (final concentration: 0.75 μg/μL).
9. Incubate for at least 24 h at 37°C/5% CO_2_ before the next major step.

**CRITICAL:** The incubation time of step 9 should be at least 24 h. Best results will be achieved after about 36 h of incubation time, but the difference in result compared to 24 h is not very big. Waiting significantly longer than 48 h will result in decreased efficiency.

### Optional: Transfection in 2D

**Timing: 1 day**

Generally, transfection in 2D uses the same steps as spheroid transfection. However, extra steps are necessary to keep YAP out of the nucleus (and thus inactivated) prior to the experiment. Cells seeded on normal well plates will have almost exclusively nuclear YAP due to the hard surface (Dupont et al., 2011). The following are two main methods to avoid nuclear YAP in 2D cell culture.

#### Serum depletion

10. Thaw cells normally as described above
11. Seed cells on 8-well slide at desired concentration (e.g., 5000 cells per well).
12. Exchange medium for DMEM without FBS and with 1% PenStrep.
13. Wait 24–48 h for cells to settle and adjust to new medium.
14. Transfect as described above and perform experiments

This method has the drawback of changing culture conditions and starving cells, which will affect their behavior and may even lead to cell death. It may be the method of choice though for cells cultured in FBS-free (and lysophosphatidic acid (LPA) and sphingosine 1-phosphate (S1P) free) medium (Yu et al., 2012). However, the protocol has not been tested yet with such cells.

#### Seeding cells on Matrigel

Cells on a thick enough layer of matrigel will not sense the stiff bottom of the well and thus YAP will be cytosolic, i.e., inactive. In this case, cells exhibit a round shape.

15. Every reagent and tool should be pre-cooled on ice.
16. Add 50 μL of matrigel to each well of an 8-well slide and carefully spread it with the tip of a pipette until the entire well is covered.
17. Incubate well plate for 30 min at 37°C/5% CO_2_.
18. Carefully seed cells on top of the gel as usual (e.g., 5000 cells per well).
19. Incubate for 24 h. Cells should be round, if viewed under a microscope.
20. Transfect as described above.

Visualization of cells on matrigel via fluorescence microscopy can become more difficult. Both due to the thick gel and due to the round shape of the cell adding more background and scattering the light.

This method is also very susceptible to cooling as gelation is reversible.

### optoYAP activation

**Timing: 3 d**

Activation of optoYAP to induce growth and invasive behavior from activated area (Figures 1C and 1D).

21. Activation of optoYAP via UV-illumination.
  a. Illuminate full well with 365 nm UV lamp for general optoYAP-activation in spheroids.
  b. Use 365 nm laser or LED and a suitable microscope for site-selective activation of optoYAP
22. Illuminate desired area of spheroid for 20 s at 0.6 mW/mm^2^ and take images of target areas for comparison
23. Incubate for 72 h at 37°C/5% CO_2_.
24. Check for growth at illuminated regions under a microscope. (troubleshooting – problems 4 and 5)

### Antibody staining of spheroids embedded in collagen gel

**Timing: 7 d**

Staining of spheroids with YAP antibodies or other antibodies of choice. Amounts are given per well of an 8-well slide.

25. Carefully remove medium with a pipette.
26. Fix spheroids with 200 μL 4% formaldehyde for 40 min in the wells of the slide.
27. Wash twice with 300 μL cold PBS for 20 min each.
28. Permeabilize cells for 20 min with 200 μL 0.5% Triton X-100 in PBS.
29. Wash with 300 μL PBS for 30 min.
30. Block with 200 μL PBS containing 1% BSA overnight (10–24 h). (troubleshooting – problem 6)
31. Dilute 1 μg primary antibodies in 200 μL PBS containing 1% BSA, add to cells and incubate for 72 h.
32. Wash twice with 300 μL cold PBS for 20 min each.
33. Incubate for 48 h with 1 μg secondary antibodies in 200 μL PBS containing 1% BSA.
34. Wash with 300 μL cold PBS for 20 min.
35. Stain with a 0.5 μg/mL solution of Hoechst in PBS for 40 min.
36. Wash with 300 μL cold PBS for 30 min.
37. Renew PBS and assure sample remains wet during storage

***Note:*** Samples are stable for months at 4°C in this state, if the sample is kept from drying out and protected from light. Prevent drying, since salt crystals from evaporated PBS will also obscure microscopy – even before the sample is entirely dried out.

***Note:*** We recommend using non-illuminated spheroids as well as non-transfected or Mock-transfected, illuminated spheroids as negative controls. As positive controls, we recommend cells on plastic substrates or spheroids transfected with YAP fused to a nuclear localization sequence.

## Expected outcomes

**Figure 2:**
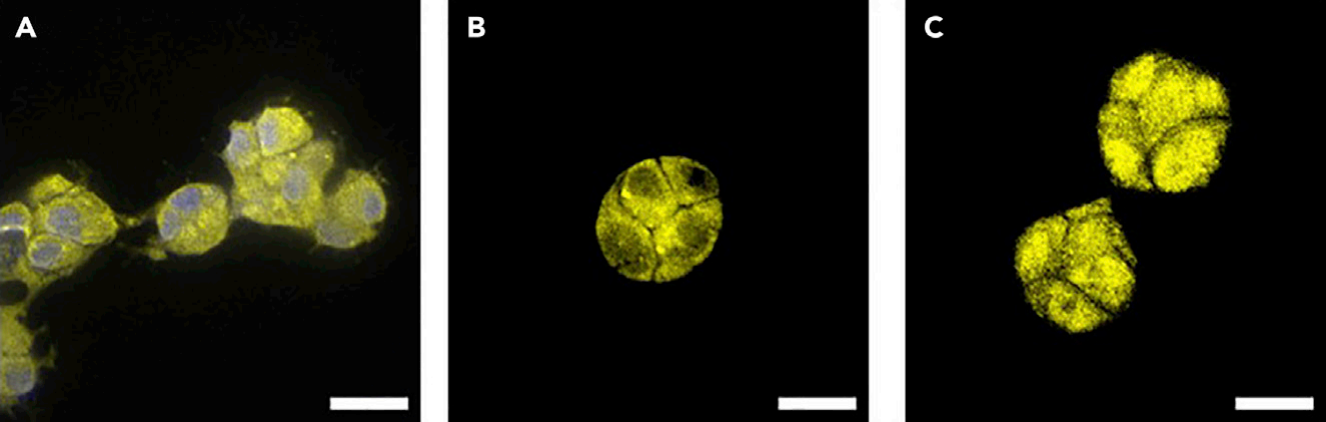
Immunofluorescence staining of YAP (A) YAP (yellow) and Hoechst (blue) staining of an invasive branch of a spheroid embedded in collagen 72 h after optoYAP activation. (B) YAP staining is cytosolic before photoactivation of optoYAP in cells on matrigel showing also their round morphology. (C) YAP staining is nuclear 4 h after activation of optoYAP in cells on matrigel. Scale bars: 20 μm. Images were acquired on a Zeiss Axiovert Observer with a Yokogawa spinning disk unit and a 63**×** oil immersion objective.

If successful, invasive growth from the activated region should be visible after incubation (see Figures 1C and 1D). Regions of the spheroid, which were not activated, should not show any signs of significant invasive growth. Negative controls (see note above) should not show invasive growth either. Furthermore, YAP (visualized with antibodies or the GFP fusion of optoYAP) should be cytosolic before activation and in non-activated regions, but nuclear in activated regions (see Figure 2). Note that several hours after activation, YAP will be cytosolic again. Thus, samples that were incubated for 72 h after activation will show cytosolic YAP. Positive controls (see note above) should have permanently nuclear YAP.

## Limitations

This protocol has been tested for a limited amount of cancer cell lines (HeLa and A431) that form stable spheroids. A successful application to other cells should be possible, but is not guaranteed and optimization would likely be required. Depending on the available equipment the activation of the optoYAP inside the spheroids may be difficult, if no sufficiently powerful lasers/LEDs in the 365 nm range are available. Furthermore, since this is not based on a multi-photon process, site-selectivity is limited to lateral selectivity with respect to the light beam due to the light beam penetrating the sample making selectivity along the light beam impossible. Additionally, the observation of the effects of optoYAP based on fluorescence imaging may be challenging, depending on the available microscopes due to the gel thickness and scattering effects obscuring the image.

## Troubleshooting

### Problem 1

Spheroids are not stable and fall apart or die shortly after transfer to the collagen gel (steps 2 and 4).

### Potential solution

Check the pH of the Collagen Type I stock solution and adjust the amount of NaOH when preparing the coating mixture accordingly. Final pH should be 7.4. If this does not solve the problem, let cells grow in low-adhesion plate for longer time periods.

### Problem 2

**Figure 3:**
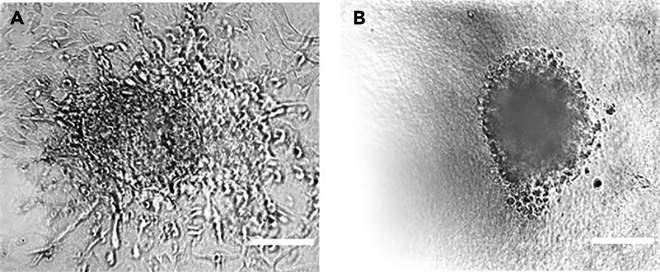
Brightfield images of damaged spheroid (A) Example of a spheroid too close to the bottom of the slide, which disintegrates. (B) Example of a spheroid damaged during embedment in collagen. Scale bars: 100 μm. Images were acquired on a Nikon eclipse Ti-E microscope with a 25**×**, 1.1. N.A. water immersion objective.

Spheroids are too close to the bottom of the well in the gel and show invasion along the bottom of the well even without activation (Figure 3A; steps 2 and 4).

### Potential solution

Collagen matrix may be not viscous enough. Check collagen concentration and pH. If this does not fix the problem, you can keep the gel on ice for some time before adding cells to enhance stiffness of the gel.

### Problem 3

Spheroids appear damaged or are too close to the bottom of the gel (Figures 3A and 3B; steps 3 and 4).

### Potential solution

Depending on the goal of the experiment it can be important to change the procedure when adding the spheroids to the collagen gel. Careful mixing will lead to a more homogenous gel, but will also usually lead to the spheroids being closer to the bottom and can cause damage. If the spheroids should be very far from the bottom and damage to them is a concern, not mixing the gel after spheroid addition can be beneficial as the spheroids will usually be farther from the bottom of the well and less damaged albeit at the cost of a more inhomogeneous gel.

### Problem 4

No visible growth after activation (step 24).

### Potential solution

Check transfection efficiency via green fluorescence of optoYAP. If needed, optimize parameters of transfection protocol. Also check cells for viability and possible contaminations.

### Problem 5

No activation visible upon illumination (step 24).

### Potential solution

Depending on gel thickness, illumination source and specifically material of the slide, it may be necessary to remove the lid of the slide before illumination from top as the activating UV light might lose too much power otherwise.

### Problem 6

Spots in fluorescence microscopy images (step 30).

### Potential solution

Replace existing BSA solution with freshly prepared solution as agglomerated BSA gives spotty signals.

## Resource availability

### Lead contact

Further information and requests for resources and reagents should be directed to and will be fulfilled by the lead contact, Hanna Engelke (hanna.engelke@uni-graz.at).

### Materials availability

All unique reagents (optoYAP plasmid) associated with this protocol are available from the lead contact.

### Data and code availability

No code was used in this protocol. Further data associated with this protocol can be found in the associated publication (Illes et al., 2021) and obtained from the lead author upon reasonable request.
